# Serial monitoring of circulating tumour DNA on clinical outcome in myelodysplastic syndromes and acute myeloid leukaemia

**DOI:** 10.1002/ctm2.1349

**Published:** 2023-07-25

**Authors:** Xinping Zhou, Wei Lang, Chen Mei, Yanling Ren, Liya Ma, Lingxu Jiang, Li Ye, Gaixiang Xu, Yingwan Luo, Lixia Liu, Shanbo Cao, Jiayue Qin, Hongyan Tong

**Affiliations:** ^1^ Department of Hematology, The First Affiliated Hospital Zhejiang University School of Medicine Hangzhou China; ^2^ Zhejiang Provincial Key Laboratory of Hematopoietic Malignancy Zhejiang University Hangzhou China; ^3^ Zhejiang Provincial Clinical Research Center for Hematological disorders Hangzhou China; ^4^ Zhejiang University Cancer Center Hangzhou China; ^5^ Department of Medical Affairs Acornmed Biotechnology Co., Ltd. Tianjin China

Dear Editor,

Diagnostic bone marrow (BM) DNA and matched plasma‐derived circulating tumour DNA (ctDNA) demonstrated excellent correlations in myelodysplastic syndromes (MDS) and acute myeloid leukaemia (AML),^1^, ^2^, ^3^, ^4^ and dynamic ctDNA monitoring contributes to predict relapse in MDS and AML patients undergoing allogeneic hematopoietic stem cell transplantation (allo‐HSCT).^5^ However, few researches are reported on the application of ctDNA dynamic monitoring assessments during the whole disease course in MDS and AML.^1^ Here, we assessed the feasibility and utility of ctDNA as a novel and minimally invasive biomarker based on targeted next‐generation sequencing (NGS) to monitor treatment outcome, track clonal evolution and predict survival. To the best of our knowledge, we firstly demonstrated the application of ctDNA concentration as an effective prognostic biomarker in MDS and AML.

The details of clinical data and methods are shown in Tables S1–S3. Thirty‐five adult patients were involved in our study. Twenty‐seven patients had pre‐ and post‐treatment plasma‐derived ctDNA, and paired baseline BM DNA NGS assessments were included for both concordance analysis and dynamic ctDNA analysis. Four patients with only baseline BM DNA and plasma‐derived ctDNA NGS assessments were used for concordance analysis. Three patients with only pre‐ and post‐treatment ctDNA assessments and one patient with only post‐treatment ctDNA assessments were used for dynamic ctDNA analysis (Figure 1).

**FIGURE 1:**
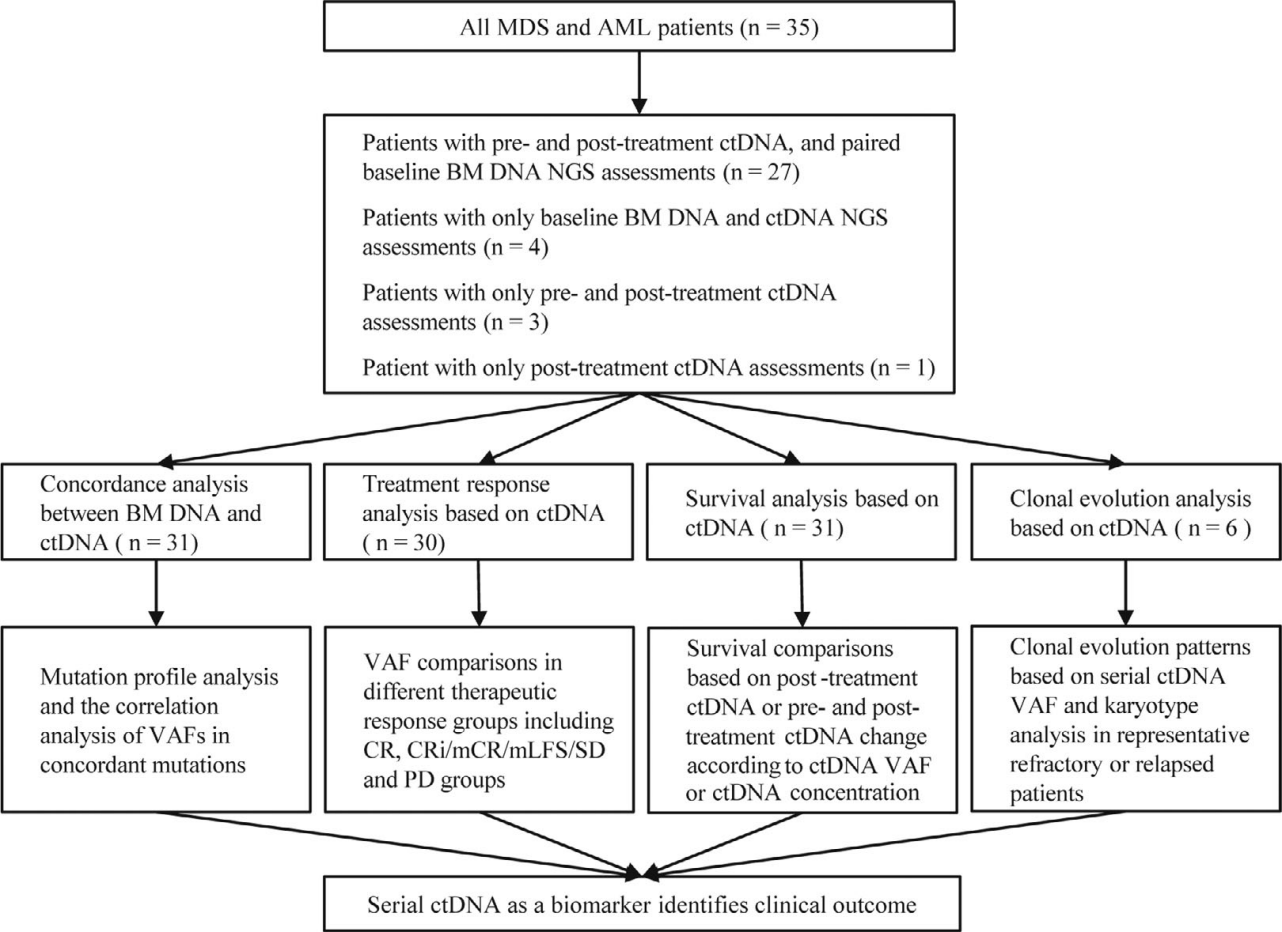
Flow diagram of study design, showing the samples and patients analyzed in the study. MDS, myelodysplastic syndromes; AML, acute myeloid leukaemia; BM, bone marrow; VAF, variant allele frequency; CR, complete response; CRi, CR with incomplete hematologic recovery; mCR, morphologic CR; mLFS, morphologic leukaemia‐free state; SD, stable disease; PD, progressive disease.

A total of 46 mutated genes with 135 mutations were detected in BM DNA and plasma‐derived ctDNA, involving 42 genes with 100 mutations (74.1%) detected both in BM DNA and plasma‐derived ctDNA (Figure 2A, Figure S1). Among the 100 concordant mutations, the concordance of variant allele frequencies (VAFs) assessment based on plasma and BM was high (*R* = .854 *p* < .001) (Figure 2B). Comparing pre‐treatment mean ctDNA concentrations with blasts in BM at baseline measured by cytological analysis, we found pre‐treatment mean ctDNA concentrations were strongly associated with BM blasts at baseline (*R* = .618, *p* < .001) (Figure 2C), indicating mean ctDNA concentration were related to tumour burden. We also reached a moderate correlation between pre‐treatment mean ctDNA VAF and BM blasts (*R* = .533, *p* = .001) (Figure S2).

**FIGURE 2:**
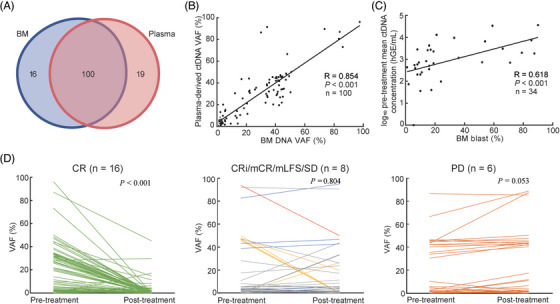
Mutation profile and treatment response analysis. (A) Numbers of concordant mutations and unique mutations identified in matched BM DNA and plasma‐derived ctDNA. (B) The correlation of VAFs in concordant mutations between BM DNA and plasma‐derived ctDNA. (C) Correlation between pre‐treatment mean ctDNA concentrations and BM blasts at baseline. (D) Comparison of pre‐ and post‐treatment VAFs based on plasma‐derived ctDNA assessments. VAFs were compared in different therapeutic response groups with different colored lines, including CR, CRi/mCR/mLFS/SD, and PD groups. CRi (*n* = 1, yellow), mCR (*n* = 1, blue), mLFS (*n* = 1, red) or SD (*n* = 5, gray). BM, bone marrow; VAF, variant allele frequency; MDS, myelodysplastic syndromes; AML, acute myeloid leukaemia; CR, complete response; CRi, CR with incomplete hematologic recovery; mCR, morphologic CR; mLFS, morphologic leukaemia‐free state; SD, stable disease; PD, progressive disease.

For patients with pre‐ and post‐treatment paired samples, dynamic change of VAFs was analyzed. Patients were segregated into three groups, including those achieved a complete response (CR), those with CR with incomplete hematologic recovery (CRi) (for AML), morphologic CR (mCR) (for MDS)/morphologic leukaemia‐free state (mLFS) (for AML) or stable disease (SD) (for MDS) and those with progressive disease (PD). Sixteen patients with CR had dramatically decreased VAFs (median VAF, 16.7% pre‐treatment vs. 0% post‐treatment, *p* < .001). Persistent VAFs were observed in CRi, mCR, mLFS and SD patients (median VAF, 3.6% pre‐treatment vs. 3.3% post‐treatment, *p* = .804). VAFs from PD patients tended to increase (median VAF, 4.7% pre‐treatment vs. 6.7% post‐treatment, *p* = .053) (Figure 2D). The post‐treatment VAFs were lower in patients with CR versus those without CR (median VAF, 0% vs. 3.7%, *p* < .001) (Figure S3A). Similarly, delta VAFs from pre‐treatment to post‐treatment were greater in patients with CR versus those without CR (median delta VAF, 12.8% vs. −.3%, *p* < .001) (Figure S3B).

As expected, post‐treatment ctDNA positivity was associated with both shorter progression‐free survival (PFS, median PFS, 5.6 vs. not reached [NR] months, *p* < .001) (Figure 3A) and overall survival (OS, median OS, 11.0 vs. NR months, *p* < .001) (Figure 3B). In the meantime, increased mean ctDNA VAF (defined as increasing post‐treatment compared with pre‐treatment ctDNA VAF) predicted decreased PFS (median PFS, 2.8 vs. 25.0 months, *p* < .001) (Figure 3C) and OS (median OS, 8.1 vs. 16.8 months, *p* = .014) (Figure 3D). Furthermore, we discovered patients with increased mean ctDNA concentration have poorer PFS (median PFS, 2.8 vs. NR months, *p* < .001) (Figure 3E) and OS (median OS, 7.9 vs. NR months, *p* < .001) (Figure 3F). However, the significant prognostic impact of pre‐treatment ctDNA status on PFS and OS with mean ctDNA VAF (Figure S4A,B) or mean ctDNA concentration (Figure S4C and D) was not discovered, indicating pre‐treatment ctDNA status was not a good biomarker to stratify the prognosis. To investigate the prognostic differences between mean ctDNA VAF and mean ctDNA concentration change from post‐ to pre‐treatment, we compared the discrimination ability of the prognostic model with the concordance index (C‐index). The model based on mean ctDNA concentration change showed better discrimination of PFS with C‐index value of .79, compared to mean ctDNA VAF with C‐index value of .75. The area under curve (AUC) for predicting 1‐year PFS indicated more excellent conformity based on ctDNA concentration change, compared to mean ctDNA VAF (AUC, .84 vs. .82) (Figure 3G). Similarity was also discovered in OS analysis (C‐index, .70 vs. .67; AUC, .78 vs. .72) (Figure 3H).

**FIGURE 3:**
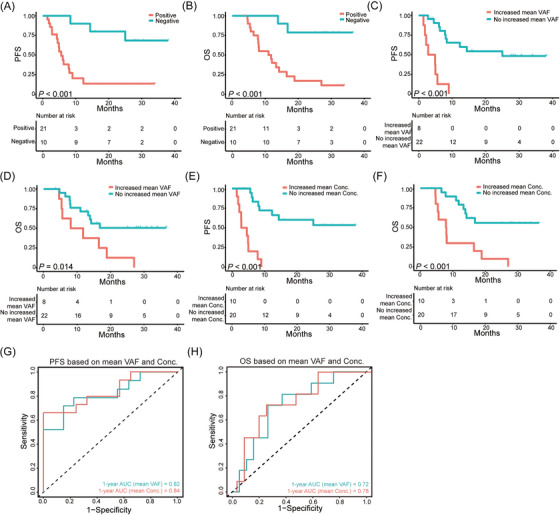
Prognostic impact of ctDNA status on survival. (A and B) PFS and OS of patients with positive ctDNA versus patients with negative ctDNA after treatment, respectively. (C and D) PFS and OS of patients with increased mean ctDNA VAF versus patients with no increased mean ctDNA VAF based on pre‐ and post‐treatment ctDNA change, respectively. (E and F) PFS and OS of patients with increased mean ctDNA Conc. versus patients with no increased mean ctDNA Conc. based on pre‐ and post‐treatment ctDNA change, respectively. (G and H) Discrimination ability of PFS and OS with receiver operating characteristic curve based on mean ctDNA VAF and mean ctDNA Conc., respectively. The sum of these points is located on the total point axis and the line is drawn downward to the survival axis to determine the likelihood of 1‐year PFS or 1‐year OS. PFS, progression‐free survival; OS, overall survival; VAF, variant allele frequency; Conc., concentration; AUC, area under curve.

Next, we tried to explore clonal evolution patterns in refractory or relapsed MDS and AML. We observed two patterns of progression from MDS to secondary AML (sAML). Patient 1 (P1) showed a linear pattern, where successive clones occurred within the previous parental clone. The acquisition of *FLT3* mutation occurred during disease relapse and subsequently transformation to AML (Figure 4A). P7 and P8 showed a branched pattern (Figure 4B, Figure S5A, respectively). For P7, the original clone harboring *PTPN11* mutation was suppressed while a new clone carrying *FLT3*‐ITD mutation expanded, leading to AML transformation. However, we only discovered one linear pattern in refractory or relapsed AML. Three representative patients (P11, P26 and P35) achieved CR and relapsed within 6−7 months (Figure 4C, Figure S5B,C, respectively). The founding clone in the primary tumour in P11 contained mutations in *WT1*, *GATA2* and *RAD21* that were all recurrent at relapse. A new subclone occurred and evolved to become the dominant clone at relapse by acquiring additional *SETD2* mutations. Compared with flow cytometry measurable residue disease results, we found that ctDNA molecular residue detection was earlier about 4 months.

**FIGURE 4:**
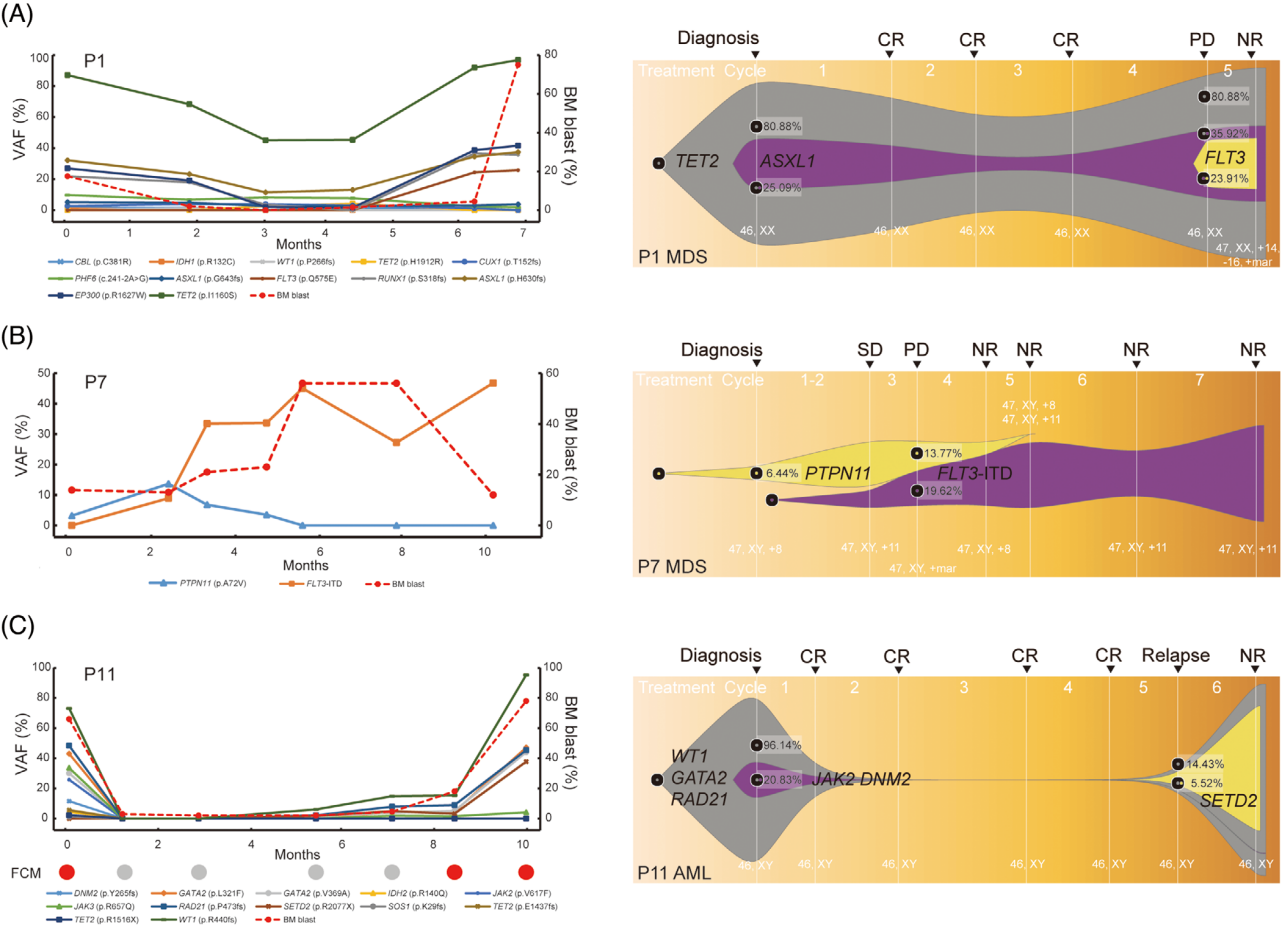
Clonal evolution patterns based on VAF and karyotype analysis in three representative refractory or relapsed patients. The patients included two MDS (A and B) and one AML patients (C). For FCM results, red and gray circle represent residue and non‐residue, respectively. VAF, variant allele frequency; MDS, myelodysplastic syndromes; AML, acute myeloid leukaemia; FCM, flow cytometry.

In summary, our study revealed that serial plasma‐derived ctDNA assessments can reflect treatment response, survival, and clonal evolution in adult MDS and AML as a minimally invasive method, which warrant the prospective use of ctDNA as a biomarker in disease monitoring.

## CONFLICT OF INTEREST STATEMENT

The authors declare that they have no competing interests.

## FUNDING INFORMATION

The National Natural Science Foundation of China Grant Number: 81800121; Zhejiang Medical Association Clinical Research Fund project, Grant Number: 2017ZYC‐A14.

## Supporting information

Supporting Information

## Data Availability

The data used in the present study are available from the corresponding author upon reasonable request.
