# Supplementary material for: Serial monitoring of circulating tumour DNA on clinical outcome in myelodysplastic syndromes and acute myeloid leukaemia
Source: Clin Transl Med. 2023 Jul 25;13(7):e1349. doi: 10.1002/ctm2.1349 (PMC10368807; doi:10.1002/ctm2.1349)
Supplement: Supplementary file 1 — Supporting Information [file CTM2-13-e1349-s001.docx]

**Supplementary Information**

**Serial monitoring of circulating tumor DNA on clinical outcome in myelodysplastic syndromes and acute myeloid leukemia**

Xinping Zhou^1,2,3,4*^, Wei Lang^1*^, Chen Mei^1,2,3,4^, Yanling Ren^1,2,3,4^, Liya Ma^1,2,3,4^, Lingxu Jiang^1^, Li Ye^1,2,3,4^, Gaixiang Xu^1,2,3,4^, Yingwan Luo^1,2,3,4^, Lixia Liu^5^, Shanbo Cao^5^, Jiayue Qin^5^, Hongyan Tong^1,2,3,4^

^1^Department of Hematology, the First Affiliated Hospital, Zhejiang University School of Medicine, Hangzhou, Zhejiang, China;

^2^Zhejiang Provincial Key Laboratory of Hematopoietic Malignancy, Zhejiang University, Hangzhou, Zhejiang, China;

^3^Zhejiang Provincial Clinical Research Center for Hematological disorders, Hangzhou, Zhejiang, China;
^4^Zhejiang University Cancer Center, Hangzhou, Zhejiang, China;

^5^Department of Medical Affairs, Acornmed Biotechnology Co., Ltd., Tianjin, China.

^*^These authors contributed equally.

**Correspondence:** Prof. Hongyan Tong, Department of Hematology, The First Affiliated Hospital, Zhejiang University School of Medicine, No. 79 Qingchun Road, Hangzhou 310003, China; e-mail: [tonghongyan@zju.edu.cn](mailto:tonghongyan@zju.edu.cn); Prof. Jiayue Qin, Department of Medical Affairs, Acornmed Biotechnology Co., Ltd., Tianjin, China. Building D4, International Enterprise Community, Changyuan Road, Wuqing District, Tianjin 301799, China; e-mail: [jyqin@live.cn](mailto:jyqin@live.cn).

This file includes:

Methods

Discussion

Figure S1 to S5

Table S1 to S3

**Methods**

**Study design**

A total of 35 patients with serial 134 plasma samples, including 21 patients with MDS and 14 patients with AML, were enrolled from the First Affiliated Hospital, Zhejiang University School of Medicine (Table S1, S2). Patients who had both BM DNA and paired plasma-derived ctDNA sequencing samples at baseline were used for concordance analysis. Those patients who had at least two serial ctDNA assessments were included in dynamic ctDNA analysis. Treatments among ctDNA assessments included hypomethylating agent (HMA), intensive chemotherapy (IC), HMA combined with chemotherapy, and best supportive care (BSC). Responses to treatment in MDS and AML were evaluated according to International Working Group (IWG) 2006 and European LeukmiaNet (ELN) 2017 AML response criteria.^1,2^ For evaluating the response of MDS patients treated with HMA, we used the best response ever achieved after at least four cycles of treatments. This study was approved by the Ethics Committee of The First Affiliated Hospital of Zhejiang University and all patients signed informed consent forms before enrollment.

**Next-generation sequencing analysis**

BM DNA and plasma-derived cell-free DNA (cfDNA) were extracted with a customized Genomic DNA kit and a customized QIAamp Circulating Nucleic Acid kit from BM and peripheral blood plasma, respectively. Gene library amplification was performed by a KAPA Hyper Prep Kit. The targeted 163-gene sequencing panel was from Acornmed Biotechnology Co. Ltd. (Table S3). Multiplex libraries were sequenced by Illumina NovaSeq instrument. To filter raw variants, the following criteria were used: average effective sequencing depth on target per sample ≥ 1000x and ≥ 2000x for DNA and cfDNA, respectively; mapping quality ≥ 30 and base quality ≥ 30; variant allele frequency (VAF) ≥ 1% for single nucleotide variation (SNV) and insertion or deletion (InDel), respectively. Reads were aligned to the human genome (version: hg19) using the Burrows-Wheeler Alignment (BWA, version 0.7.12). MarkDuplicates tool in Picard was used to mark PCR duplicates. BaseRecalibrator and IndelRealigner from Genome Analysis Toolkit (GATK, version 3.8) were used for the recalibration and realignment of the BWA data, respectively. Mutect2 was performed to identify SNV and InDel. All the variants were annotated by the ANNOVAR software, including 1000G projects, COSMIC, SIFT, and Polyphen. Reported mutation criterions, involved in the analysis, are as follows. Mutations that comply with the mutant gene mentioned in the guidelines (such as NCCN guidelines) will be reported. Furthermore, the mutations, which have been confirmed by the population database, ClinVar database or the literature itself as possibly benign, will not be reported. Mutations that have not been discovered in patients mentioned above even if functional prediction/cell assay confirms effects on function also will not be reported.

**Statistical analysis**

Statistical tests were performed using SPSS (version 22.0) or R package (version 3.5.2). Continuous variables were compared using Mann-Whitney or Wilcoxon test. Categorical variables were compared using chi-square or Fisher’s exact test. Concordance analysis was assessed using Spearman correlation analysis. Survival analysis were evaluated with the Kaplan-Meier curves using the log-rank test. Concentration of ctDNA was calculated by multiplying mean ctDNA VAF by the input concentration of cfDNA and dividing by 3.3.^3^ A two-sided *P* value < 0.05 was considered statistically significant.

**Discussion**

In this study, we performed targeted serial plasma-derived ctDNA NGS assessments to monitor tumor burden in adult MDS and AML patients during treatments. We found that molecular responses were in accordance with clinical responses. Serial ctDNA tracking provided a unique molecular signature with feasibility and utility to monitor dynamic changes of hematological disease.

Based on the sequencing data at the same gene capture area in both BM DNA and paired plasma-derived ctDNA at baseline, we found that plasma was largely in accordance with BM sequencing. The median VAF of 16 unique mutations detected in BM was 1.7 % (range, 1.1% to 5.4%), and it was 1.6% (range, 1.0% to 3.4%) in 19 unique mutations tested in plasma. In view of this, small subclonal populations with VAF < 2 % were more likely to be missed, and no significant difference in the distribution of the above mutation sites were discovered. These results suggested that BM DNA and plasma-derived ctDNA sequencing may be complementary in the sequencing assessments. Compared with mean ctDNA VAF, we discovered mean ctDNA concentration showed a stronger correlation with BM blasts (R, 0.618 vs. 0.533, respectively).

We also assessed the value of dynamic ctDNA monitor in adult MDS and AML patients. Some studies showed that molecular assessment of the BM had important significance for risk of disease relapse and OS.^4,5^ Plasma-derived ctDNA assessment as a noninvasive measure, has been suggested to be a reliable marker of residual disease in lymphoma and prognostic biomarker in MDS and AML undergoing allo-SCT.^6-8^ Our research showed that positive ctDNA or increased mean VAF can predict shorter PFS and OS. In the meanwhile, we firstly explore the application of ctDNA concentration in MDS and AML, which was well explained in lymphoma and solid tumors.^3,9-12^ We discovered post-treatment ctDNA concentration, rather than pre-treatment ctDNA concentration, was an effective biomarker to stratify the prognosis.

Serial molecular profiling of plasma-derived ctDNA in the same patient has contributed greatly to our understanding of disease progression. Previous reports have demonstrated that members of signaling pathways mutations that promoted proliferation correlate with progression towards sAML.^13,14^ Consistent with this, our study described MDS cases with lately acquired *NF1*, *FLT3*, *PTPN11* or *RAS* mutations, representing more aggressive clones, progressed to AML eventually. In addition, acquisition of these mutations could be several months prior to progression towards sAML. Similaly in relapsed AML patients, re-expansion of malignant clone, or newly acquired subclone which resulted in subsequent relapse, could be detected several months before AML relapse. For the three AML patients with continuous monitoring samples, we compared the FCM and ctDNA detection results at the same time point, and found that ctDNA was earlier than the FCM detection of disease residue in two of the three patients (P11 and P26). In another patient (P35), ctDNA was synchronized with the FCM to detect disease residue. These results suggested that regular ctDNA monitor may effectively predict adult MDS progression and AML relapse. As FCM and ctDNA testing explore disease residues at two different dimensions, the former is based on the cluster of differentiation antigen level, and the latter is based on the gene level, there might be inconsistent detection results at the same time point. In the future, big data research is needed to draw more reliable conclusions. Judging from our results, we suggest the combination of the two methods may provide new ideas for practical clinical treatment decisions.

Meanwhile, we studied clonal evolution in MDS and AML patients receiving disease modifying treatments. Dynamic ctDNA changes revealed complex clonal patterns in response to treatment. Different patterns of clonal evolution were observed, including linear pattern and branching pattern. All AML patients treated with chemotherapy showed negative ctDNA results when they achieved a CR. In contrast, in MDS patients, mutations frequently persist during HMA agent treatments even when complete morphological responses were achieved, indicating malignant clones were not eliminated by HMAs ^15-17^ and blasts percentage in morphology assessment sometimes underestimated disease burden. Mutations with low-level VAFs detected in CR might reflect persistence of mutated hematopoietic stem and progenitor cells after epigenetic therapy,^18,19^ which therefore might be potential causes of secondary resistance and disease relapse. Early clinical intervention may be helpful to prolong the survival of patients with molecular recurrence.

Our study has several limitations. First, due to the small number of adult MDS and AML patients, large-scale plasma-derived ctDNA clinical studies are needed to verify these conclusions. Second, in order to better study the clinical outcome, in vitro cytological experiments and animal models combined with ctDNA sequencing need to be studied in the future.

**References**

1. Dohner H, Estey E, Grimwade D, et al: Diagnosis and management of AML in adults: 2017 ELN recommendations from an international expert panel. Blood 129:424-447, 2017

2. Cheson BD, Bennett JM, Kantarjian H, et al: Report of an international working group to standardize response criteria for myelodysplastic syndromes. Blood 96:3671-4, 2000

3. Scherer F, Kurtz DM, Newman AM, et al: Distinct biological subtypes and patterns of genome evolution in lymphoma revealed by circulating tumor DNA. Sci Transl Med 8:364ra155, 2016

4. Klco JM, Miller CA, Griffith M, et al: Association Between Mutation Clearance After Induction Therapy and Outcomes in Acute Myeloid Leukemia. JAMA 314:811-22, 2015

5. Jongen-Lavrencic M, Grob T, Hanekamp D, et al: Molecular Minimal Residual Disease in Acute Myeloid Leukemia. N Engl J Med 378:1189-1199, 2018

6. Roschewski M, Dunleavy K, Pittaluga S, et al: Circulating tumour DNA and CT monitoring in patients with untreated diffuse large B-cell lymphoma: a correlative biomarker study. Lancet Oncol 16:541-9, 2015

7. Herrera AF, Kim HT, Kong KA, et al: Next-generation sequencing-based detection of circulating tumour DNA After allogeneic stem cell transplantation for lymphoma. Br J Haematol 175:841-850, 2016

8. Nakamura S, Yokoyama K, Shimizu E, et al: Prognostic impact of circulating tumor DNA status post-allogeneic hematopoietic stem cell transplantation in AML and MDS. Blood, 2019

9. Bohers E, Viailly PJ, Becker S, et al: Non-invasive monitoring of diffuse large B-cell lymphoma by cell-free DNA high-throughput targeted sequencing: analysis of a prospective cohort. Blood Cancer J 8:74, 2018

10. Meriranta L, Alkodsi A, Pasanen A, et al: Molecular features encoded in the ctDNA reveal heterogeneity and predict outcome in high-risk aggressive B-cell lymphoma. Blood 139:1863-1877, 2022

11. Li M, Chen J, Zhang B, et al: Dynamic monitoring of cerebrospinal fluid circulating tumor DNA to identify unique genetic profiles of brain metastatic tumors and better predict intracranial tumor responses in non-small cell lung cancer patients with brain metastases: a prospective cohort study (GASTO 1028). BMC Med 20:398, 2022

12. Pastor B, Andre T, Henriques J, et al: Monitoring levels of circulating cell-free DNA in patients with metastatic colorectal cancer as a potential biomarker of responses to regorafenib treatment. Mol Oncol 15:2401-2411, 2021

13. Bejar R: What biologic factors predict for transformation to AML? Best Pract Res Clin Haematol 31:341-345, 2018

14. Menssen AJ, Walter MJ: Genetics of progression from MDS to secondary leukemia. Blood 136:50-60, 2020

15. Unnikrishnan A, Papaemmanuil E, Beck D, et al: Integrative Genomics Identifies the Molecular Basis of Resistance to Azacitidine Therapy in Myelodysplastic Syndromes. Cell Rep 20:572-585, 2017

16. Merlevede J, Droin N, Qin T, et al: Mutation allele burden remains unchanged in chronic myelomonocytic leukaemia responding to hypomethylating agents. Nat Commun 7:10767, 2016

17. Uy GL, Duncavage EJ, Chang GS, et al: Dynamic changes in the clonal structure of MDS and AML in response to epigenetic therapy. Leukemia 31:872-881, 2017

18. Craddock C, Quek L, Goardon N, et al: Azacitidine fails to eradicate leukemic stem/progenitor cell populations in patients with acute myeloid leukemia and myelodysplasia. Leukemia 27:1028-36, 2013

19. Will B, Zhou L, Vogler TO, et al: Stem and progenitor cells in myelodysplastic syndromes show aberrant stage-specific expansion and harbor genetic and epigenetic alterations. Blood 120:2076-86, 2012

**
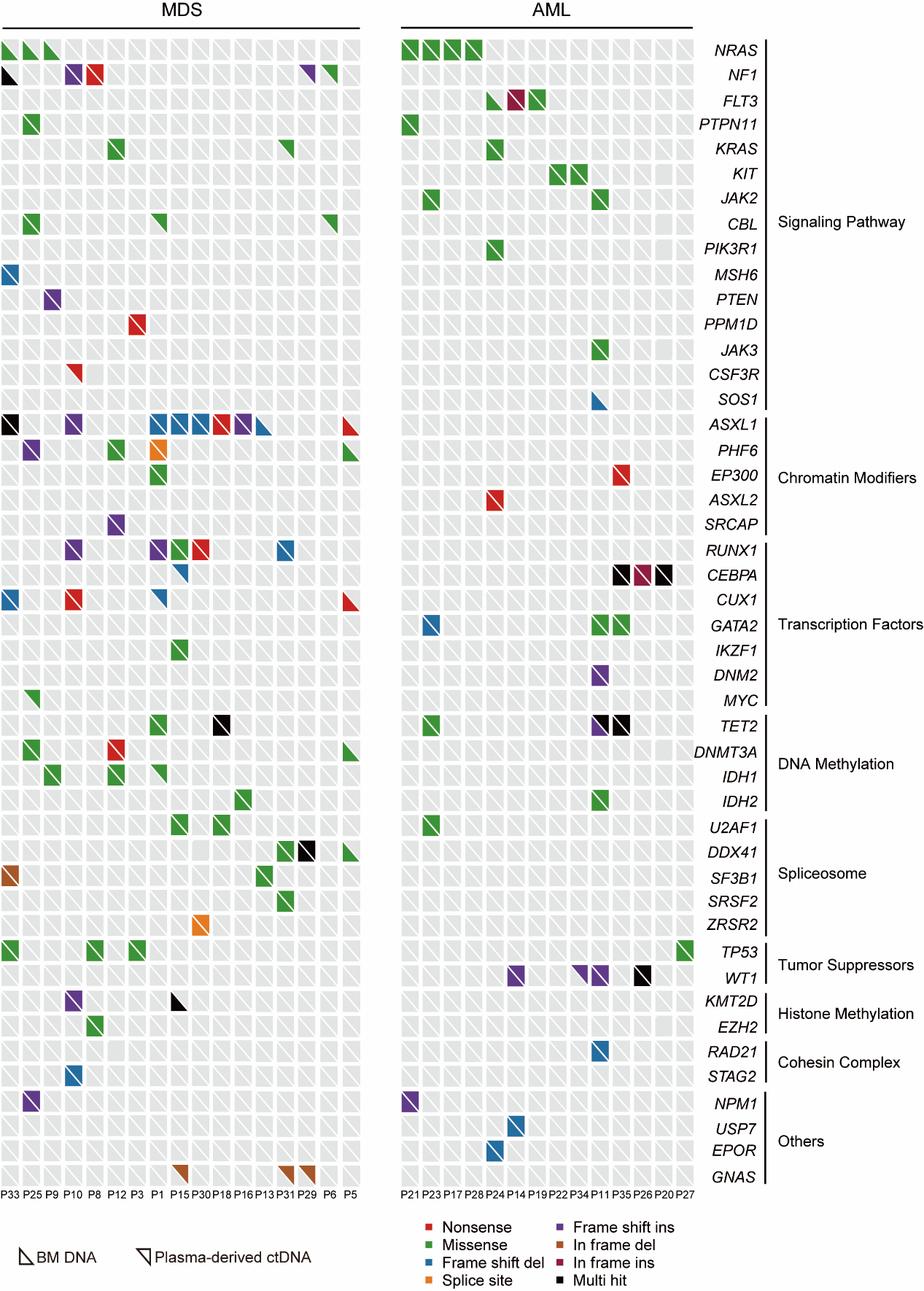
**

**Figure S1.** Mutation landscape detected in BM DNA and paired plasma-derived ctDNA of 31 adult MDS and AML patients. BM, bone marrow; MDS, myelodysplastic syndromes; AML, acute myeloid leukemia.

**
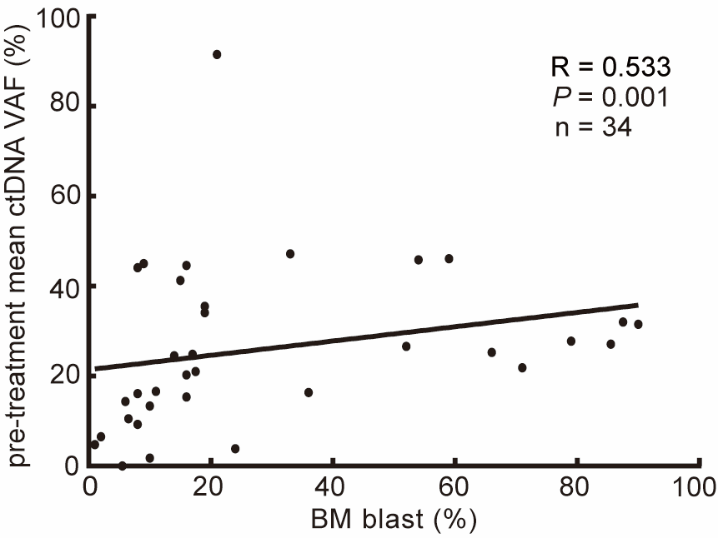
**

**Figure S2.** Correlation between pre-treatment mean ctDNA VAFs and BM blasts at baseline. VAF, variant allele frequency; BM, bone marrow.


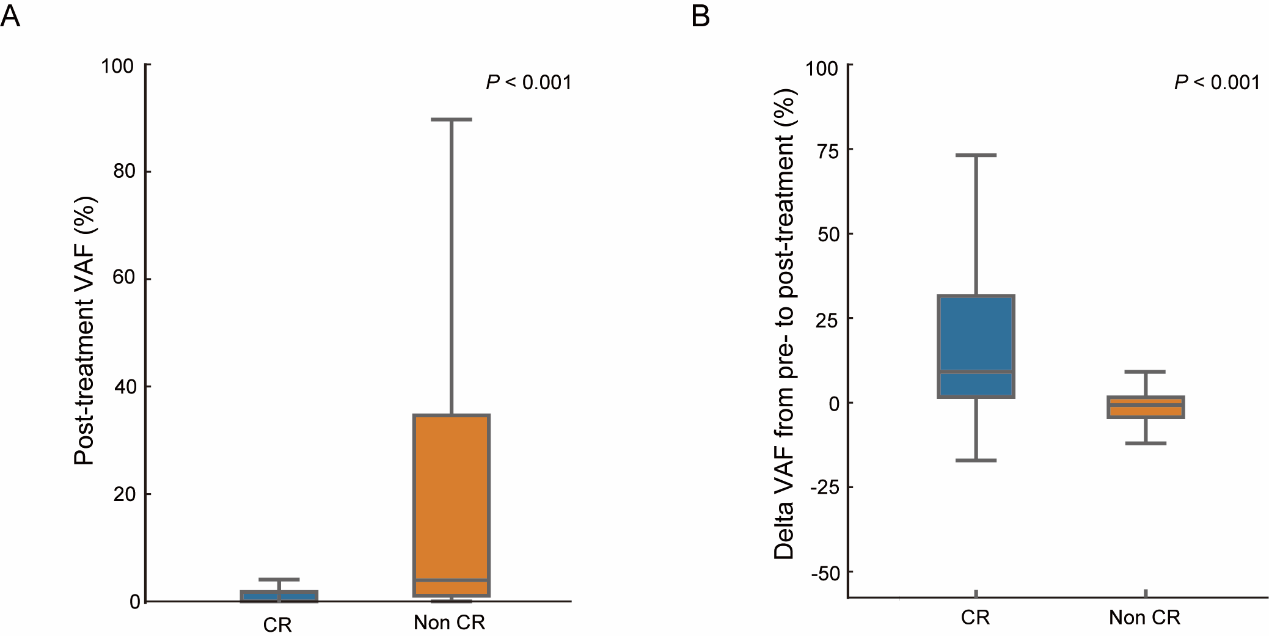


**Figure S3.** Comparison of post-treatment VAF in patients with CR versus those without CR (A) and delta VAF from pre-treatment to post-treatment in patients with CR versus those without CR (B). VAF, variant allele frequency; CR, complete remission.


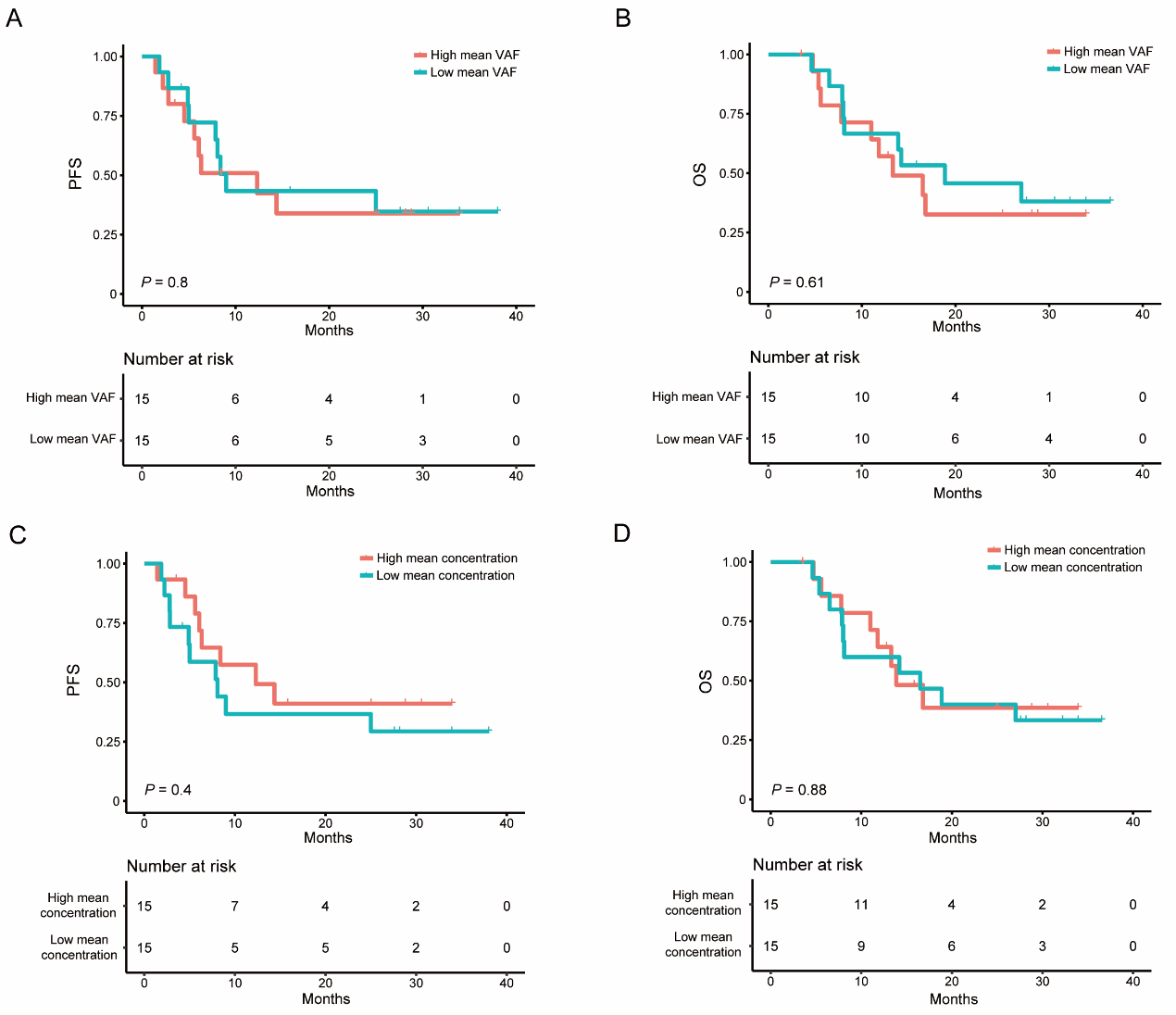


**Figure S4.** Prognostic impact of pre-treatment ctDNA status on PFS and OS based on mean ctDNA VAF (A, B) and mean ctDNA concentration (C, D), respectively. PFS, progression-free survival; OS, overall survival; VAF, variant allele frequency.


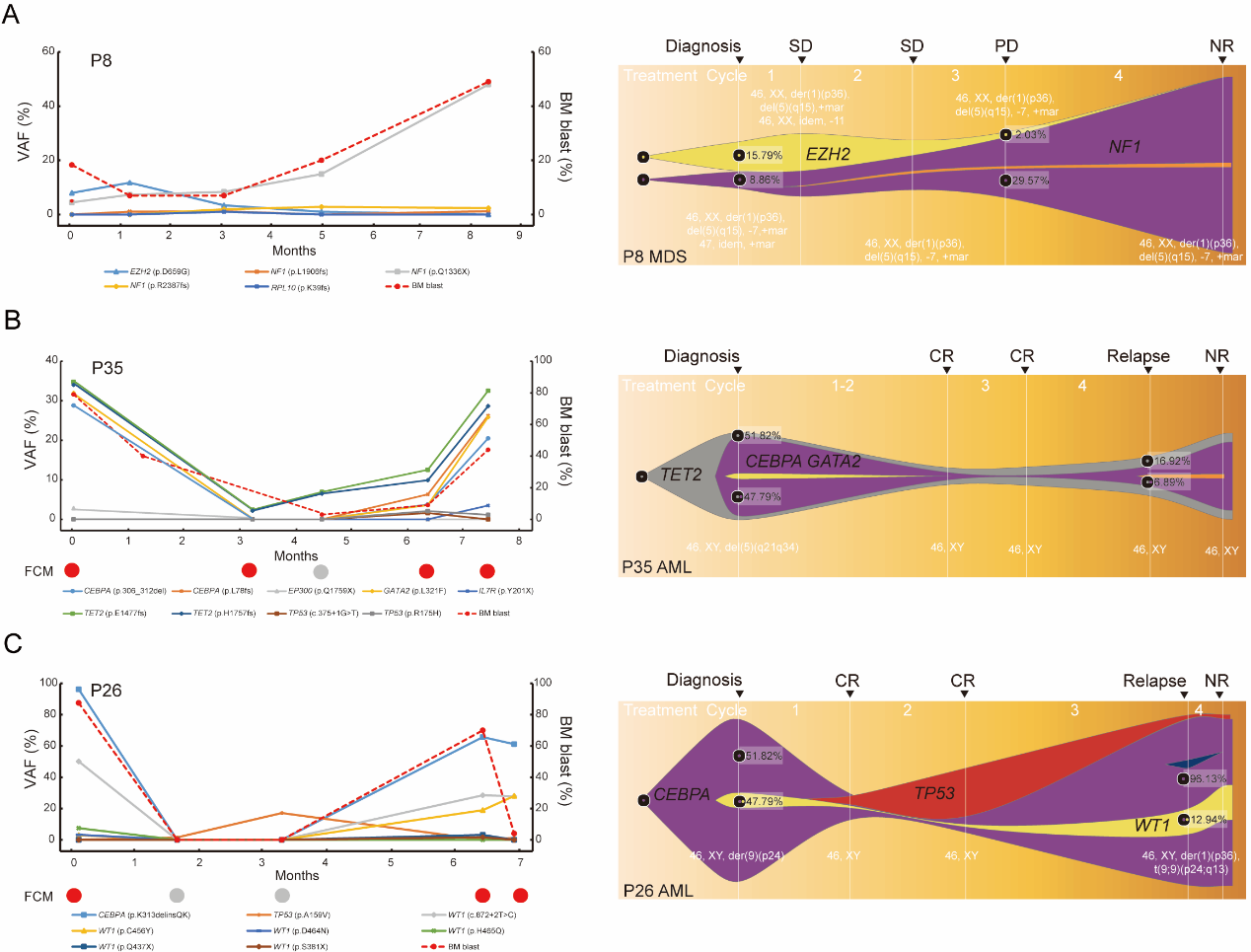


**Figure S5.** Clonal evolution patterns based on VAF and karyotype analysis in three patients, including P8 (A), P35 (B) and P26 (C). For FCM results, red and gray circle represent residue and non-residue, respectively. VAF, variant allele frequency; MDS, myelodysplastic syndromes; AML, acute myeloid leukemia; FCM, flow cytometry.

**Table S1. Baseline characteristics of 35 adult MDS and AML patients**

| Baseline characteristics | n (%) or median (range) |
| --- | --- |
| Age (years) | 61 (19-81) |
| Gender |  |
| Male, N (%) | 27 (77.1) |
| Female, N (%) | 8 (22.9) |
| WHO subtypes |  |
| MDS | 21 (60.0) |
| De novo AML | 12 (34.3) |
| Secondary AML | 2 (5.7) |
| CBC |  |
| Hemoglobin (g/dL) | 74 (46-121) |
| Platelet counts (/L) | 48 (12-596) |
| ANC (/L) | 0.9 (0.1-8.1) |
| Bone marrow blasts (%) | 16 (6.0-90.0) |
| IPSS-R (N = 21 with < 20% blasts) |  |
| Intermediate | 5 (23.8) |
| High | 11 (52.4) |
| Very high | 5 (23.8) |
| Treatment |  |
| HMA | 19 (54.3) |
| IC | 11 (31.4) |
| HMA + chemotherapy | 1 (2.9) |
| BSC | 4 (11.4) |

Abbreviations: MDS, myelodysplastic syndromes; AML, acute myeloid leukemia; CBC, complete blood count; ANC, absolute neutrophil counts; IPSS-R, revised international prognostic scoring system; HMA, hypomethylating agent; IC, intensive chemotherapy; BSC, best support care.

**Table S2. Clinical and biological features of 35 adult MDS and AML patients**

| Patient | Sex | Age at diagnosis | WHO subtypes | WBC (×10^9/L) | ANC (/L) | Platelet counts (/L) | Hemoglobin (g/dL) | Bone marrow blasts (%) | IPSS-R | Treatment | Survival |
| --- | --- | --- | --- | --- | --- | --- | --- | --- | --- | --- | --- |
| P1 | F | 61 | MDS | 4 | 1.4 | 85 | 39 | 17.5 | High | HMA + chemotherapy | Dead |
| P2 | F | 72 | MDS | 1.8 | 0.2 | 77 | 596 | 14 | High | HMA | Dead |
| P3 | M | 65 | MDS | 2.4 | 0.9 | 76 | 16 | 16 | Very high | HMA | Dead |
| P4 | M | 71 | MDS | 8.9 | 7.6 | 56 | 22 | 8 | Very high | HMA | Dead |
| P5 | M | 64 | MDS | 2.6 | 1.3 | 114 | 61 | 15.5 | Intermediate | HMA | Alive |
| P6 | M | 58 | MDS | 1.4 | 0.1 | 91 | 93 | 11 | High | BSC | Dead |
| P7 | M | 61 | MDS | 3.9 | 2 | 46 | 67 | 11 | Very high | HMA | Dead |
| P8 | F | 67 | MDS | 2.1 | 0.5 | 98 | 57 | 19 | Very high | HMA | Dead |
| P9 | M | 81 | MDS | 1.1 | 0.3 | 112 | 81 | 16 | High | HMA | Alive |
| P10 | M | 78 | MDS | 0.7 | 0.2 | 71 | 156 | 14 | High | HMA | Dead |
| P11 | M | 61 | de novo AML | 0.8 | 0.3 | 121 | 33 | 66 | NA | IC | Dead |
| P12 | F | 78 | MDS | 0.8 | 0.3 | 121 | 33 | 16 | High | HMA | Alive |
| P13 | F | 59 | MDS | 2.3 | 0.9 | 74 | 591 | 8 | High | HMA | Alive |
| P14 | M | 29 | de novo AML | 1.7 | 0.3 | 51 | 107 | 36 | NA | IC | Alive |
| P15 | M | 55 | MDS | 4 | 2.6 | 109 | 48 | 11 | High | HMA | Alive |
| P16 | M | 64 | MDS | 2.1 | 1.1 | 72 | 144 | 15 | High | HMA | Dead |
| P17 | M | 19 | de novo AML | 63.1 | 8.1 | 49 | 18 | 54­ | NA | IC | Alive |
| P18 | M | 38 | MDS | 3.6 | 0.7 | 71 | 12 | 19 | Very high | HMA | Dead |
| P19 | F | 23 | de novo AML | 5.9 | 1.1 | 57 | 47 | 90 | NA | IC | Alive |
| P20 | M | 72 | de novo AML | 6.1 | 1.2 | 100 | 81 | 71 | NA | IC | Dead |
| P21 | M | 27 | de novo AML | 24.2 | 6.7 | 65 | 45 | 61 | NA | IC | Alive |
| P22 | F | 50 | de novo AML | 22.4 | 3.4 | 118 | 278 | 71 | NA | IC | Alive |
| P23 | M | 64 | de novo AML | 37.7 | 7.5 | 50 | 126 | 59 | NA | IC | Alive |
| P24 | M | 50 | de novo AML | 2.9 | 2.4 | 49 | 13 | 17 | NA | IC | Alive |
| P25 | M | 58 | MDS | 4.4 | 2.6 | 70 | 125 | 6 | Intermediate | BSC | Dead |
| P26 | M | 63 | de novo AML | 99.1 | 2.1 | 80 | 22 | 87.5 | NA | IC | Dead |
| P27 | M | 69 | sAML | 0.9 | 0.2 | 70 | 28 | 21 | NA | HMA | Dead |
| P28 | M | 52 | de novo AML | 5.3 | 0.6 | 73 | 93 | 33 | NA | IC | Alive |
| P29 | M | 76 | MDS | 3.3 | 2.1 | 116 | 28 | 6 | Intermediate | HMA | Alive |
| P30 | M | 52 | MDS | 1.5 | 0.6 | 66 | 45 | 8 | High | HMA | Alive |
| P31 | F | 63 | MDS | 1 | 0.4 | 100 | 44 | 6.5 | Intermediate | BSC | Alive |
| P32 | M | 66 | MDS | 1.1 | 0.24 | 118 | 45 | 9 | Intermediate | HMA | Dead |
| P33 | M | 71 | MDS | 1.03 | 0.29 | 54 | 27 | 10 | High | BSC | Dead |
| P34 | M | 57 | sAML | 0.65 | 0.12 | 67 | 54 | 24 | NA | HMA | Dead |
| P35 | M | 61 | de novo AML | 33.6 | 3.49 | 79 | 73 | 79 | NA | HMA | Dead |

Abbreviations: MDS, myelodysplastic syndromes; AML, acute myeloid leukemia; sAML, secondary AML; F, female; M, male; CBC, complete blood count; ANC, absolute neutrophil counts; IPSS-R, revised international prognostic scoring system; HMA, hypomethylating agent; IC, intensive chemotherapy; BSC, best support care; NA, Not Applicable.

**Table S3. The 163-gene targeted next-generation sequencing panel**

| **Gene** | **Transcript** | **Pathway** |
| --- | --- | --- |
| *ABCB1* | NM_000927 | Drug Metabolism |
| *ABCG2* | NM_001257386 | Drug metabolism |
| *ABL1* | NM_005157 | Signaling Pathway |
| *ADSL* | NM_000026 | Drug metabolism |
| *ALK* | NM_004304 | Signaling Pathway |
| *ANKRD26* | NM_001256053 | Others |
| *ARID1B* | NM_017519 | Epigenetics-related Genes |
| *ASXL1* | NM_015338 | Chromatin Modifiers |
| *ASXL2* | NM_018263 | Chromatin Modifiers |
| *ATM* | NM_000051 | DNA Damage Response |
| *ATRX* | NM_000489 | Epigenetics-related Genes |
| *B2M* | NM_004048 | Immune Escape |
| *BCL11B* | NM_138576 | Transcription Factors |
| *BCL2* | NM_000633 | Others |
| *BCL6* | NM_001706 | Transcription Factors |
| *BCOR* | NM_001123383 | Epigenetics-related Genes |
| *BCORL1* | NM_021946 | Epigenetics-related Genes |
| *BIRC3* | NM_001165 | Apoptosis-related Genes |
| *BLM* | NM_000057 | DNA Damage Response |
| *BMP7* | NM_001719 | Signaling Pathway |
| *BRAF* | NM_004333 | Signaling Pathway |
| *BTK* | NM_000061 | Signaling Pathway |
| *CALR* | NM_004343 | Others |
| *CBL* | NM_005188 | Signaling Pathway |
| *CCND3* | NM_001760 | Cell-cycle Regulation |
| *CD79A* | NM_001783 | Signaling Pathway |
| *CDA* | NM_001785 | Drug metabolism |
| *CDKN1B* | NM_004064 | Cell-cycle regulation |
| *CDKN2A* | NM_000077 | Cell-cycle regulation |
| *CEBPA* | NM_004364 | Transcription Factors |
| *CEP72* | NM_018140 | Drug metabolism |
| *CPA2* | NM_001869 | Drug metabolism |
| *CREBBP* | NM_004380 | Epigenetics-related genes |
| *CRLF2* | NM_022148 | Signaling Pathway |
| *CSF3R* | NM_156039 | Signaling Pathway |
| *CTCF* | NM_006565 | Epigenetics-related genes |
| *CTLA4* | NM_005214 | Drug metabolism |
| *CTNNB1* | NM_001904 | Signaling Pathway |
| *CUX1* | NM_181552 | Transcription Factors |
| *CYP2B6* | NM_000767 | Others |
| *CYP2C19* | NM_000769 | Others |
| *CYP2C8* | NM_000770 | Others |
| *CYP3A4* | NM_017460 | Others |
| *CYP3A5* | NM_000777 | Drug metabolism |
| *DARS* | NM_145507 | Drug metabolism |
| *DDX41* | NM_016222 | Spliceosome |
| *DHX15* | NM_001358 | Spliceosome |
| *DHX30* | NM_138615 | Spliceosome |
| *DIS3* | NM_014953 | Others |
| *DKC1* | NM_001363 | Telomere Maintenance |
| *DNM2* | NM_001005361 | Transcription Factors |
| *DNMT3A* | NM_022552 | DNA Methylation |
| *DOK5* | NM_001294161 | Drug metabolism |
| *ELANE* | NM_001972 | Others |
| *EP300* | NM_001429 | Chromatin Modifiers |
| *EPOR* | NM_000121 | Others |
| *ERCC1* | NM_001166049 | Drug metabolism |
| *ETNK1* | NM_018638 | Cell Metabolism |
| *ETV6* | NM_001987 | Transcription Factors |
| *EZH2* | NM_001203247 | Histone Methylation |
| *FAM46C* | NM_017709 | Others |
| *FAT1* | NM_005245 | Others |
| *FBXW7* | NM_033632 | Signaling Pathway |
| *FCGR3A* | NM_000569 | Drug metabolism |
| *FLT3* | NM_004119 | Signaling Pathway |
| *FOXO1* | NM_002015 | Transcription Factors |
| *GATA1* | NM_002049 | Transcription Factors |
| *GATA2* | NM_032638 | Transcription Factors |
| *GATA3* | NM_002051 | Transcription Factors |
| *GFI1* | NM_005263 | Transcription Factors |
| *GNAS* | NM_080425 | Others |
| *GSTM1* | NM_000561 | Drug metabolism |
| *GSTP1* | NM_000852 | Drug metabolism |
| *HAX1* | NM_006118 | Others |
| *HLA-DRB1* | NM_002124 | Others |
| *ID3* | NM_002167 | Others |
| *IDH1* | NM_001282386 | DNA Methylation |
| *IDH2* | NM_002168 | DNA Methylation |
| *IKZF1* | NM_006060 | Transcription Factors |
| *IL7R* | NM_002185 | Signaling Pathway |
| *IMPDH2* | NM_000884 | Drug metabolism |
| *ITPA* | NM_001267623 | Drug metabolism |
| *JAK1* | NM_002227 | Signaling Pathway |
| *JAK2* | NM_004972 | Signaling Pathway |
| *JAK3* | NM_000215 | Signaling Pathway |
| *KDM5C* | NM_004187 | Epigenetics-related genes |
| *KDM6A* | NM_001291415 | Epigenetics-related genes |
| *KIT* | NM_000222 | Signaling Pathway |
| *KMT2A* | NM_001197104 | Epigenetics-related genes |
| *KMT2C* | NM_170606 | Epigenetics-related genes |
| *KMT2D* | NM_003482 | Histone Methylation |
| *KRAS* | NM_004985 | Signaling Pathway |
| *MAP2K1* | NM_002755 | Signaling pathway |
| *MED12* | NM_005120 | Signaling pathway |
| *MPL* | NM_005373 | Signaling pathway |
| *MSH6* | NM_000179 | Signaling Pathway |
| *MTHFR* | NM_001330358 | Drug metabolism |
| *MTRR* | NM_001364440 | Drug metabolism |
| *MYC* | NM_002467 | Transcription Factors |
| *MYD88* | NM_002468 | Signaling pathway |
| *NF1* | NM_000267 | Signaling Pathway |
| *NFATC2* | NM_001136021 | Drug metabolism |
| *NOTCH1* | NM_017617 | Signaling Pathway |
| *NPM1* | NM_002520 | Histone Methylation |
| *NR3C1* | NM_000176 | Transcription Factors |
| *NRAS* | NM_002524 | Signaling Pathway |
| *NSD2* | NM_133330 | Epigenetics-related genes |
| *NT5C2* | NM_012229 | Cell Metabolism |
| *NUDT15* | NM_001304745 | Drug metabolism |
| *PAX5* | NM_016734 | Transcription Factors |
| *PDGFRA* | NM_006206 | Signaling Pathway |
| *PDGFRB* | NM_002609 | Signaling Pathway |
| *PHF6* | NM_001015877 | Chromatin Modifiers |
| *PIGA* | NM_002641 | Cell Metabolism |
| *PIK3R1* | NM_181523 | Signaling Pathway |
| *PLCG2* | NM_002661 | Signaling pathway |
| *PNPLA3* | NM_025225 | Drug metabolism |
| *PPM1D* | NM_003620 | Signaling Pathway |
| *PRPF8* | NM_006445 | Spliceosome |
| *PRPS1* | NM_002764 | Cell Metabolism |
| *PTEN* | NM_000314 | Signaling Pathway |
| *PTPN11* | NM_002834 | Signaling Pathway |
| *RAD21* | NM_006265 | Cohesin Complex |
| *RB1* | NM_000321 | Cell-cycle regulation |
| *RPL10* | NM_006013 | Ribosome-related Genes |
| *RRM1* | NM_001033 | Drug metabolism |
| *RRM2* | NM_001034 | Drug metabolism |
| *RRM2B* | NM_001172477 | Drug metabolism |
| *RUNX1* | NM_001754 | Transcription Factors |
| *SERPINE1* | NM_000602 | Drug metabolism |
| *SETBP1* | NM_015559 | Epigenetics-related genes |
| *SETD2* | NM_014159 | Epigenetics-related genes |
| *SF3B1* | NM_012433 | Spliceosome |
| *SH2B3* | NM_005475 | Signaling Pathway |
| *SLC22A1* | NM_003057 | Drug metabolism |
| *SLCO1A2* | NM_001386878 | Drug metabolism |
| *SLCO1B1* | NM_006446 | Drug metabolism |
| *SMC1A* | NM_006306 | Cohesin complex |
| *SMC3* | NM_005445 | Cohesin complex |
| *SOD2* | NM_000636 | Drug metabolism |
| *SOS1* | NM_005633 | Signaling Pathway |
| *SPI1* | NM_003120 | Transcription Factors |
| *SRCAP* | NM_006662 | Chromatin Modifiers |
| *SRP72* | NM_006947 | Others |
| *SRSF2* | NM_003016 | Spliceosome |
| *STAG2* | NM_001042749 | Cohesin Complex |
| *STAT3* | NM_003150 | Signaling Pathway |
| *STAT5B* | NM_012448 | Signaling Pathway |
| *TERT* | NM_198253 | Others |
| *TET2* | NM_001127208 | DNA Methylation |
| *TNF* | NM_000594 | Drug metabolism |
| *TNFAIP3* | NM_006290 | Signaling Pathway |
| *TNFRSF14* | NM_003820 | Signaling Pathway |
| *TP53* | NM_000546 | Tumor Suppressors |
| *TPMT* | NM_000367 | Others |
| *U2AF1* | NM_006758 | Spliceosome |
| *UGT1A1* | NM_000463 | Others |
| *UGT1A8* | NM_019076 | Others |
| *USH2A* | NM_206933 | Others |
| *USP7* | NM_003470 | Others |
| *WT1* | NM_024426 | Tumor Suppressors |
| *XRCC5* | NM_021141 | Drug metabolism |
| *ZRSR2* | NM_005089 | Spliceosome |
